# A Study of Leptin Serum Concentrations in Patients with Major Beta-Thalassemia

**Published:** 2013-04-22

**Authors:** I Shahramian, E Akhlaghi, A Ramezani, A Rezaee, N Noori, E Sharafi

**Affiliations:** 1 Assistant Professor of Pediatric, Department of Pediatrics, Zabol University of Medical Sciences, Zabol , Iran.; 2 Medical Student, Zabol University of Medical Sciences, Zabol , Iran.; 3 Department of Healht, Zabol University of Medical Sciences, Zabol , Iran.; 4 Department of Biochemisterey, Zabol University of Medical Sciences, Zabol , Iran.; 5 Professore of Pediatric Cardiology, Pediatric Cardiology, Children and Adolescent Health Research Center, Zahedan University of Medical Sciences, Zahedan, Iran.; 6 Resident of Ophthalmology, Ophtalmology Research center, Zahedan University of Medical Sciences, Zahedan, Iran.

**Keywords:** Beta-Thalassemia, Leptin, Ferritin, Child

## Abstract

**Background:**

The aim of this study was to evaluate leptin serum levels in patients with major beta thalassemia which was also associated with their ferritin serum levels.

**Materials and Methods:**

This case-control study was performed on 90 children -6 months to 16 years old, in Zabol, Amir- al- Momenin Hospital. Patients were divided in two groups and were matched in age and sex. All Children were examined and those eligible children who had not known heart disease, iron deficiency anemia, kidney disease, diabetes, fever and systemic diseases were enrolled after taking the informed consent of their parents. After collecting the samples, leptin and ferritin levels of the serum were measured in two groups by ELISA method. Then, the data was analyzed by the related statistical tests and SPSS 20 software.

**Results:**

The mean of the serum levels of leptin and ferritin showed a significant difference in the case and control groups (P-value<0.05). An inverse statistical correlation was found for the serum levels of leptin and ferritin among the studied groups (P-value<0.05). Levels of leptin in the case group showed a significant gender difference (P-value<0.05), while based on BMI and age, no significant difference was observed for the serum levels of leptin in the case group.

**Conclusion:**

Based on the results of this study, major thalassemia reduces serum levels of leptin regardless of age and body mass. The study also found an inverse statistical correlation between serum levels of leptin and ferritin among the studied people.

## Introduction

Every year, 100,000 neonates are born with hemoglobinopathies around the world. Thalassemia is the most common heterogeneous disease of the human being. It is a disease of high prevalence in Mediterranean, Indian, North Chinese, and Pacific populations. Recently, the quantity and quality of the life of these patients have been significantly improved by regular transfusion and iron chelating therapy (1, 2).

Leptin, a polypeptide of 146 amino acids, is an OB gene product, which is widely produced in different tissues, including bone marrow and Adipocyte ( also known as lipocytes and fat cells, are the cells that primarily compose adipose tissue, specialized in storing energy as fat) and has a direct correlation with body mass index (3, 4). This hormone is multi-functional and leads to increase energy levels and affects angiogenesis, inflammation, and hematopoiesis (5-7). It is regulated by an intricate complex which includes glucocorticoids, thyroid and insulin.

Bone marrow contains active OB genes as well as fat cells. The fat content of bone marrow in human regulates his/ her hematopoiesis activity (8-9). The structural similarity between the leptin receptor and the cytokines signaling molecules cell signaling of the hematopoiesis system improves the growth potential of the active hormones in form of blood cells (10-13). In addition, leptin binds to the hematopoietic CDNA receptor of humans and the destruction of leptin receptor stimulates the defected production of erythrocyte products (8). Consequently, due to mutations in the major beta-thalassemia, several breaks occur in Process of red blood cells maturation and antagonized with some complications such as osteoporosis, hepatosplenomegaly, hypothyroidism and insulin resistance ( 14-20) , and based on the important role of vascular chronic inflammation in the pathophysiology of complications in thalassemia, the most crucial factors for creating these implication are hemolysis, impaired erythropoiesis and inflammation (21-22) .

Due to the effectiveness of leptin on vascular inflammation, high levels of leptin correlate with the increased endothelial inflammatory response in beta-thalassemia. On the other hand, the pituitary dysfunction of the hypophysis - hypothalamus axis leads to endocrine disorders in patients with thalassemia, and the presence of leptin on the hypophysis-hypothalamus axis as well as its deficiency leads to persistent immaturity of the hypophysis-hypothalamus function.

In fact, the ferritin level is associated with iron level, so that the iron level is estimated by ferritin level. However, during blood transfusion therapy in thalassemia patients, associated with an increase in iron resulting in an increased oxidative stress and tissue damage. Lipid peroxide damage markers are including the melon-dialdehyde–antioxidant enzymesupeode dismutase nitric. These markers are also significantly associated with serum ferritin level. And their increase will lead to increased ferritin level of serum.

Ferritin reflects the iron storage of body with the normal value of 150 to300 μg/l. Here, as a result of iron overload, we face an increase of ferritin level in thalassemia patients (24-25). Studies on relationship between ferritin and leptin have suggested a possible link which is independent of its relationship with BMI and inflammation. These studies suggested that iron overload may lead to a decrease in leptin level (3). Chronic hemolysis can also increase platelet and erythrocyte adhesions to endothelial cells and therefore, increases in iron amounts have an important role in damage and inflammation of blood vessels. Following the above- mentioned cases and the possible association between the serum levels of leptin and ferritin, the current study was conducted to analyze the serum level of leptin in patients with major thalassemia.

## Materials and Methods

In this case-control study, forty- five children with an age range between 6 to 16 years old, suffering from thalassemia, diagnosis by hemoglobin electrophoresis. These children referred to Zabol Amir- al- Momenin Hospital for receiving Packed Red Blood Cell. On the other hand, 45 healthy children of the same age range were also recruited who were referred to the same hospital for the review is for articles. They were matched in terms of age with the thalassemia patients.

All Children were examined and those eligible children who had not known heart disease, iron deficiency anemia, kidney disease, diabetes, fever and systemic diseases were enrolled in this study after taking the informed consent of their parents.

From all of the children, 5 ^cc^blood was drawn at 8:00 am. Samples were centrifuged at 3000 g for 10 minutes at 5 ° C. Separated serum was kept at -70 fridges till measurement time of ferritin and leptin. Finally, under the cold chain, it was transferred to the Biochemistry Lab of Zabol University of Medical Sciences. Then, 250 micron was isolated from serum samples in order to analyze ferritin by ELISA method/kit (US) and the other serum samples used for evaluation of leptin level by ELISA method Kit (Canada).

Height and weight were measured in all children. The recumbent length for children under 2 years were graded using a flat wooden table and weight measurements for children under 2 years were performed by using balance weights Mika with difference of 100gr. Then BMI was calculated with (W _Kg_ / H^m²^) (weight in kilograms divided by height to the power of 2) formula.

**Statistical Analysis**

Data was collected using the SPSS software version 20, and descriptive statistics were Performed by statistical independent t-test, ANOVA and P <0.05 was considered as the significance level.

## Results

In this study, 90 cases of children admitted to the pediatric department of Amir-al- Momenin hospital, Zabol were divided into two groups: 45 patients as a case group (children with major beta thalassemia receiving a packed cell) and 45 Children as controls among all studied children, 74.6% were male and 25.4% were female. Their average age was 10.16 ±4.25. The BMI mean of patients was16.630±2.201. The Mean of the serum leptin levels in the case and control groups were 1.277 ±2. 22 and 8.169 ± 12.244, respectively.

The comparison of the mean serum levels of leptin in the control and case groups showed a significant difference (P-value <0.05). The results are shown in Table I. The mean of the serum ferritin level in the case group was 683.206 ±156. 126 and in the control group was 107.496 ±123. 851. The comparison of the mean of the serum ferritin in both case and control groups showed a significant difference (P-value <0.05). Comparison of serum leptin levels showed a significant statistical difference between male and female in case group (P-value=0.03) (P-value=0.525). 

BMI and age- based comparisons of serum leptin levels in both groups did not show a significant difference. On the other hand, age- based comparison of serum leptin level in both groups no showed a significant difference (P-value=0.067). The data is shown in table II.

In this study which was conducted to evaluate leptin in patients with beta-thalassemia, serum leptin levels were significantly lower than normal participants. Ferritin level was also much higher in pediatric patients than normal cases, so that a significant relationship between serum levels of leptin and ferritin was observed. 

**Table I:** Comparison of the mean serum level of leptin& ferritin. This table shows a significant difference between the case and control groups about the leptin and ferritin.

	**Control**		**Case**		**P** **-value**
	**Mean**	**SD**		**SD**	
**Leptin**	1.277	2.224	P=0.001	12.244	
**Ferritin**	638.206	156.12	P=0.001	123.851	

**Table II:** Comparison of serum leptin levels in the case group based on sex, BMI and age. This table showed a significant relationship between sex and leptin serum level, and no significant relationship between body mass index (BMI), age and leptin serum level.

**Variable**		**Mean**	**SD**	**P-value**
**Sex**	**Male**	0.507	0.438	P=0.03
	**Female**	2.081	3.005	
**BMI**	<15	1.032	1.98	P=0.525
	15-19	1.167	1.76	
	>19	2.083	4.08	
**Age**	<5	0.266	0.276	P=0.067
	5-10	0.500	0.493	
	>10	1.963	2.82	

## Discussion

In this study which was conducted to evaluate leptin in patients with beta-thalassemia, serum leptin levels in patients with thalassemia was significantly lower than normal participants. 

Moreover, studies performed by Dr. Choobineh ,Karachali of and Dr. Hamdolallah Karamifar illustrated that leptin serum level was lower in major thalassemia patients compared to healthy controls (3,23,26). In fact, iron over load followed by iron deposition in fat cells can lead to toxic effects of iron which is the result of free radical formation and inhibits the activity of adiposities.

Along with the destruction of the fat cell membrane and the dysfunction in adipose tissue, it leads to a decrease in leptin serum level (15). 

The leptin receptor is available on the bone marrow cells, hemathopoic and stem cells. In hemolytic anemia such as major beta-thalassemia, this defect in the hematopoietic cells may lead to reduced levels of leptin in these patients.

In the present study, serum ferritin level in patients with major thalassemia was higher than normal Patients. In studies conducted by Nadeem Ikram et al, and Piperno A et al serum ferritin level in patients were higher than normal children (24). Actually, ferritin serum level increases with severity in major Beta- thalassemia.

In this study, an inverse relationship was found between serum leptin and ferritin. In a study by Nikolas Chaliasos et al on oedipal cytokine serum and vessel inflammatory markers’ level in major beta-thalassemia, a significant inverse correlation was found between serum levels of leptin and ferritin (22).

Also, in a study by Joseph Scott et al on the effects of iron on regulation of adiponectin and leptin, it was stated that there is an inverse significant relationship between BMI- free serum leptin and ferritin levels (25); thus, fat cell in thalassemia patients is unable to produce leptin, partly due to the toxic effects of increased iron and ferritin levels, and the result of this increase in ferritin is reduced level of leptin.

Leptin is regulated by problematical complexes such as insulin and thyroid .In the patients with major thalassemia, following ferritin increase and iron deposition in these tissues, occurred hypothyroidism and insulin resistance and from this time, increase in ferritin level lead to leptin level reduction.

In the present study, there was a significant correlation between serum leptin and sex genders in such a way that leptin were more in females than males. In a study by G.V.Z. Dedousiss et al on inverse relationship between plasma levels of leptin and receptor level of transferring solvent in patients with thalassemia, leptin was higher in women than men. This is due to the less mRNA expression of leptin in men compared to women. The second reason is that sex hormones have an important role in the regulation of leptin. Also; BMI in women is more than men resulting in an increased production of leptin in women than in men (8).

In the present study, there was no significant relationship between BMI based-leptin level and age-based leptin. However, in a study by Dr.Choobineh et al to evaluate leptin level, there was no association between leptin and BMI, but it had a significant relationship with age (3). In another study by Suzuki and his colleagues on the relationship between obesity and serum markers of oxidative stress and inflammation in Japan, the relationship between leptin serum and fat mass was studied and it was concluded that an increase in fat mass can lead to an increase in leptin production (27).

However, there is no relationship between BMI and leptin in the present study and it is probably due to the fact that 73% of the studied thalassemia patients had a BMI = 15to19. These people did not have any increased BMI and fat, so we did not expect to observe increased leptin. Here, contrary to our expectation, there was no increase in BMI with increasing age. Therefore, increase of age could not result in an increase of fat mass as well as leptin production. Thus, no significant relationship was found between leptin and age.

## Conclusion

Given the significant difference in leptin levels in both case and control groups and based on the fact that leptin was much lower in the case group than in the control group as well as its effects on hematopoiesis, the endocrine system and the hypophisis- hypothalamus axis, it can be concluded that low levels of leptin have a role in the development of complications in thalassemia and this association is independent of age and body mass, but it is associated with sex.
